# 47461 Regulation of the immune response in the tumor microenvironment of lung adenocarcinoma

**DOI:** 10.1017/cts.2021.438

**Published:** 2021-03-30

**Authors:** Glenn Simmons

**Affiliations:** University of Minnesota Medical School

## Abstract

ABSTRACT IMPACT: This work will provide a rational approach to improve the efficacy of current immunotherapy approaches in patients that have historically responded poorly to immune checkpoint inhibitors. OBJECTIVES/GOALS: Recent evidence of immunogenic cell death as a predictor of response to therapy has increased the interest in monitoring the presence of damage-associated molecular pattern protein (DAMPs). By regulating DAMP expression, our lab is interested in discovering new ways to improve the patient response rate to immune checkpoint inhibition. METHODS/STUDY POPULATION: Using cultured cell, and a limited number of patient tumors and serum (n=4), we measured intracellular and extracellular levels of DAMP molecule, high mobility group box 1 (HMGB1) using enzyme-linked immunosorbent assays and immunoblots. Immunological assayed were compared to the expression of immune checkpoint molecules PD-1/PDL1 on patient tumors as presented in pathology reports. RESULTS/ANTICIPATED RESULTS: HMGB1 release was associated with increased levels of PD-L1 on tumor cells. Targeted inhibition of HMGB1 altered the expression of programmed death-ligand 1 (PD-L1), a target for immune checkpoint inhibition therapy. Patients with higher levels of PD-L1 possessed increased levels of HMGB1 in serum. DISCUSSION/SIGNIFICANCE OF FINDINGS: This implies that regulating the expression of HMGB1 could have an effect on the response of patients to immunotherapy. The main objective of the work is to determine the potential benefit of targeting HMGB1 to improve the efficacy of current therapeutic approaches to treating lung cancer.

